# Crack Detection Method for Wind Turbine Tower Bolts Using Ultrasonic Spiral Phased Array

**DOI:** 10.3390/s24165204

**Published:** 2024-08-11

**Authors:** Hongyu Sun, Jingqi Dong, Xi Diao, Xincheng Huang, Ziyi Huang, Zhichao Cai

**Affiliations:** 1 School of Physical Sciences and Engineering, Beijing Jiaotong University, Beijing 100044, China; shymanuscript@163.com (H.S.); 23341053@bjtu.edu.cn (J.D.); 23126638@bjtu.edu.cn (X.D.); 20271183@bjtu.edu.cn (X.H.); 2 State Key Laboratory of Performance Monitoring and Protecting of Rail Transit Infrastructure, East China Jiaotong University, Nanchang 330013, China; 3 Department of Electrical and Electronic Engineering, University of Nottingham, University Park, Nottingham NG7 2RD, UK; eeyzh3@nottingham.ac.uk

**Keywords:** ultrasonic phased array, Fermat spiral array, crack detection, wind turbine bolts, non-destructive testing

## Abstract

High-strength bolts are crucial load-bearing components of wind turbine towers. They are highly susceptible to fatigue cracks over long-term service and require timely detection. However, due to the structural complexity and hidden nature of the cracks in wind turbine tower bolts, the small size of the cracks, and their variable propagation directions, detection signals carrying crack information are often drowned out by dense thread signals. Existing non-destructive testing methods are unable to quickly and accurately characterize small cracks at the thread roots. Therefore, we propose an ultrasonic phased array element arrangement method based on the Fermat spiral array. This method can greatly increase the fill rate of the phased array with small element spacing while reducing the effects of grating and sidelobes, thereby achieving high-energy excitation and accurate imaging with the ultrasonic phased array. This has significant theoretical and engineering application value for ensuring the safe and reliable service of key wind turbine components and for promoting the technological development of the wind power industry.

## 1. Introduction

Wind power, as a form of clean, stable, and economical new energy, is currently recognized as one of the most technologically mature, scalable, and commercially viable primary energy sources in the development of new power systems. It plays a crucial role in achieving China’s “carbon peak and carbon neutrality” goals and the transition to low-carbon energy [1,2,3]. The “Global Wind Energy Report 2023”, released by the Global Wind Energy Council (GWEC), indicates that to maximize the utilization of wind energy resources and further enhance the power generation capacity of wind turbines, the tower heights and rated power of advanced wind turbines worldwide are increasing each year. The overall wind power industry and wind turbine manufacturing technology are experiencing vigorous development. However, large wind power bases are mainly distributed in areas with abundant wind resources, facing extreme environments such as rain, snow, storms, dust, and low temperatures year-round, resulting in very harsh operating conditions. Moreover, with continuous increases in the manufacturing heights of super-large wind turbines, the tall towers that directly support large components, such as rotors and nacelles, must withstand higher-intensity alternating wind loads. This makes the critical components more prone to fatigue failure, which could even lead to catastrophic accidents, such as wind turbine collapses. Therefore, it is necessary to conduct timely inspections and accurate assessments of the key components of in-service wind turbine towers.

**Figure 1:**
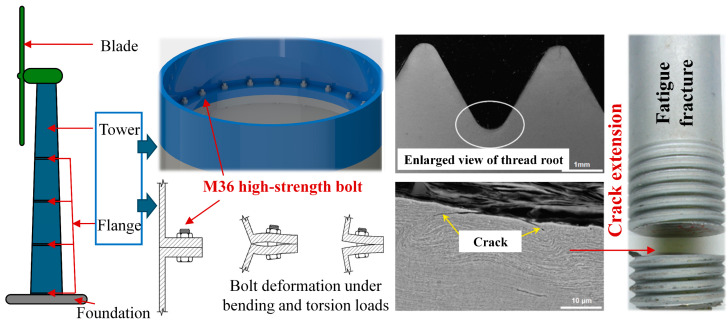
Schematic diagram of high-strength bolt connections and fatigue defects at the thread root in wind turbine towers.

As shown in Figure 1, high-strength bolts are key connecting components of the wind turbine tower. These bolts are subjected to gravity loads, dynamic alternating air loads, and loads from the start–stop operations of the turbine over long periods. The thread roots of these high-strength bolts, which have significant axial pre-tightening force, are prone to stress concentration and the initiation of fatigue cracks. These cracks can lead to fatigue failure along the circumference of the bolts, potentially causing the tower to bend or even collapse, leading to major safety incidents. Therefore, to ensure the safety of equipment and personnel during the service period of wind turbines, it is necessary to regularly detect the fatigue damage of the tower bolts.

Common bolt detection methods include eddy current testing [4], magnetic particle testing [5], and ultrasonic testing [6], among others. Eddy current testing is particularly sensitive to circumferential surface cracks near the surface of the bolts and offers advantages such as fast detection speed and easy automation. However, due to the structural complexity at the junction of the nut and bolt, as well as the thread root [7], it is extremely difficult to collect defect signals in these areas during eddy current testing. Additionally, issues such as signal lag during detection limit the further application of eddy current testing technology in complex bolt structures [8]. Magnetic particle testing requires completely cleaning the entire bolt, especially the threaded part [9], making the process cumbersome and inefficient. Furthermore, when axially magnetizing the bolt at the end, the magnetic field direction is parallel to the threads, making it difficult to effectively detect circumferential cracks within the threads. Thus, for the disassembly detection of in-service bolts, eddy current and magnetic particle testing methods are unlikely to achieve ideal results.

Ultrasonic testing, with its strong penetration capability, high detection efficiency, and ability to be implemented online, is widely used for the non-destructive testing of wind power bolts. Dong M. S. et al. [10] conducted ultrasonic testing on thread root cracks in bolts, and the results showed that ultrasonic testing could effectively detect and quantify cracks as small as 0.5 mm wide. Wagle S. and Kato H. [11] used ultrasonic testing methods to detect cracks in bolts used for aluminum alloy connections, effectively identifying small cracks within the bolts based on echo intensity. Pan Q. et al. [12] analyzed the impact of bolt stress on ultrasonic echo signals and established a corresponding quantitative evaluation model. Lee J. H. [13] and Chen J. Z. [14] studied the ultrasonic phased array detection method for bolts. However, using longitudinal wave fan scanning requires manual 180° rotation of the probe, and the detection distance is limited to three near-field zones. This makes it unable to detect cracks at the far end of the bolt and results in low detection efficiency. Moreover, existing ultrasonic testing methods face challenges such as deformation waves and interference waves caused by repeated action on the bolt’s variable cross-section contour boundary, making crack echo signal identification difficult. Additionally, some bolts have protruding heads or small end faces that prevent probe movement, leading to limited space for in-service detection, inconvenient operation, and poor accessibility of the ultrasonic probe [15].

Ultrasonic phased array testing technology uses computer control to manage the excitation sequence of array elements in various transducer arrays, achieving beam focusing and steering. This process completes beam synthesis and characteristic regulation, ultimately enabling scanning imaging [16]. Compared to traditional ultrasonic testing, ultrasonic phased array technology offers multi-angle scanning and the ability to inspect complex material structures, providing advantages in accuracy, reliability, real-time performance, and intuitiveness. The spatial distribution characteristics of the excited sound field are closely related to the arrangement of the array elements. Currently, the element arrangements for phased array ultrasonic transducers can be broadly classified into three modes: periodic arrays, random arrays, and spiral arrays [17]. In a periodic array, the elements are arranged at equal intervals according to a certain pattern. The periodic array structure of focusing ultrasonic transducers includes various types, such as linear arrays, rectangular arrays, and concentric circular arrays, based on the arrangement of the elements. Random arrays have no specific pattern for element arrangement, whereas spiral arrays arrange elements according to a spiral equation, allowing for dense element arrangements [18]. Research indicates that spiral arrays have lower levels of grating lobes and sidelobes compared to periodic arrays, and higher energy at the focal point compared to random arrays [19].

In summary, ultrasonic phased array testing technology achieves precise control of the sound beam through delay control of the array elements, offering excellent beam directionality. However, current phased array periodic array detection methods, primarily using linear, planar, and ring array element structures, have relatively sparse and uniformly spaced element distributions, making it difficult to achieve high levels of sound field energy and beam purity with the excited ultrasound. Therefore, this paper proposes a novel Fermat spiral array structure tailored to the structural characteristics of wind turbine tower bolts. By utilizing the compactness and non-periodicity of ultrasonic array elements arranged along a Fermat spiral, the filling rate of the phased array is significantly increased without enlarging the overall size of the phased array, thereby reducing the levels of grating and sidelobes. This approach provides better imaging for cracks originating at the thread roots, enabling online ultrasonic phased array detection of wind turbine tower bolt cracks under high-energy pure sound field excitation. Additionally, the method can be readily adapted to inspect various other bolts used in different industries, including critical bolts in aircraft structures and engines, the integrity of bolts in engine components and suspension systems, and high-strength bolts used in bridges, buildings, and other infrastructures.

## 2. Simulation Model

### 2.1. Subsection

**Figure 2:**
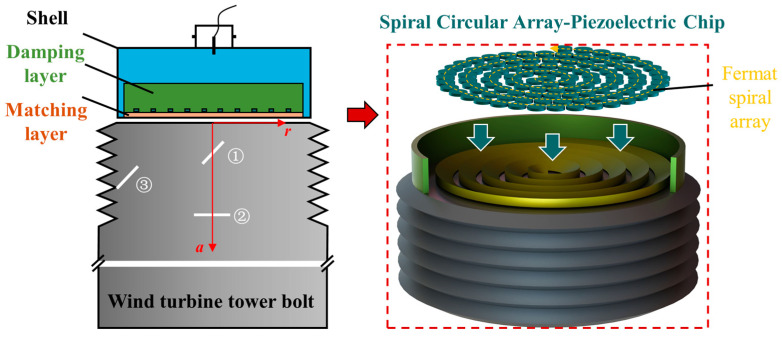
Three-dimensional schematic diagram of the basic structure, crack defects, and array element arrangement of the ultrasonic spiral phased array probe for wind turbine tower bolts.

The sidelobes and grating lobes of the ultrasonic radiation sound field can be effectively suppressed by a random array. However, traditional random arrays have a low fill factor, making it difficult to achieve high sound field intensity. In contrast, spiral arrays can significantly increase the fill factor while achieving a compact arrangement of array elements. The Fermat spiral is an equiangular spiral, with its polar equation given by: *r*^2^ = *θb*^2^. Here, *b* is the spiral coefficient, which directly affects the pitch and fill rate; in this work, it is fixed at a value of 3. A coefficient of 3 provided a balance between a high fill rate, minimal element overlap, and acceptable grating lobe levels for our specific bolt geometry and target resolution. Figure 2 shows a schematic diagram of the geometric model of the designed spiral phased array probe. The array elements of the phased array (circular array elements in this case) were arranged along the Fermat spiral, with the center of the circular array coinciding with the spiral. During the arrangement process, each array element was closely arranged tangentially along the spiral.

Figure 2 shows a cross-section of the spiral phased array probe and the bolt, indicating the positions of three defects. Defect ① is located on the central axis of the bolt, with its center on the plane of the second thread root and a tilt angle of 45° relative to the *r*-axis. Defect ② is also located on the central axis of the bolt, with its center on the plane of the sixth thread root and parallel to the *r*-axis. Defect ③ is positioned at the fourth thread root on the left side, with a tilt angle of 45° relative to the *r*-axis.

### 2.2. Simulation Model

The construction of the simulation model in this paper was completed using the Solid Mechanics module in COMSOL Multiphysics 6.2. The excitation of the piezoelectric array elements was achieved through the application of external loads. Boundary conditions in the normal direction were set to acoustic absorption to prevent boundary reflection waves from affecting the detection signal. The high-strength 42CrMo M36 bolts of the wind turbine tower were defined as linear elastic materials. The emission and reception of the spiral array were obtained through the loading and measurement of body loads. The control equation used in the simulation is as follows:

(1) $$\rho \frac{\partial^{2}u}{\partial t^{2}}=\nabla \cdot \left(S_{inel}+C:\varepsilon_{el}\right)+F_{V}$$

where ***u*** is the displacement vector, *t* is time, *ρ* is the mass density, and ***F***_V_ is the body load. In addition, S_inel_ is the elastic entropy, ***C*** is the 4th order elasticity tensor, “:” is the double contraction, and the elastic strain *ε*_el_ is the difference between the total strain and all inelastic strains. Therefore, according to the aforementioned control equation, the particle displacement distribution within the entire computational domain, which represents the ultrasonic wave propagation process, can be simulated and solved by applying specific loads to the material. The parameters used in the simulations are presented in Table 1.

Subsequently, phased array inspection data of the bolts containing defects were collected using the methods of individual element emission and full array reception. Based on the principle of delay and sum, the entire spiral phased array is focused on all the preset pixel points in the image. The number of elements in the phased array was set to *N*, the element spacing was set to *u*, the number of pixel points between adjacent elements was set to *N_e_*, the propagation speed of the sound wave in the test object was set to *v*, the number of samples per element was set to *N_s_*, and the sampling frequency was set to *f_s_*. The full matrix data are denoted by *St* and *r*(*ns*). The transmission distance of the sound wave from the transmitting element to the pixel point was calculated, and the signal finally returned to the receiving element as *E*_t_ → *p* → *E*_r_.

(2) $$d_{t,r}\left(a,z\right)=\left[\sqrt{{\left(a_{t}-a\right)}^{2}+z^{2}}+\sqrt{{\left(a_{r}-a\right)}^{2}+z^{2}}\right]\cdot \frac{u}{N_{e}}$$

The propagation delay was as follows:

(3) $$T_{t,r}=\frac{d_{t,r}\left(a,z\right)}{v}$$

The value *I*[*p*] of pixel *p* in the TFM imaging image was obtained as follows:

(4) $$I\left[p\right]=\sum_{t=0}^{N-1}\sum_{r=0}^{N-1}S_{t,r}\left[T_{t,r}\left(a,z\right)\cdot f_{s}\right]\frac{\omega_{t,r}\left(a,z\right)}{d_{t,r}\left(a,z\right)}$$

where *ω_t_*_,*r*_(*a*,*z*) represents the apodization function [20].

## 3. Results and Discussions

**Figure 3:**
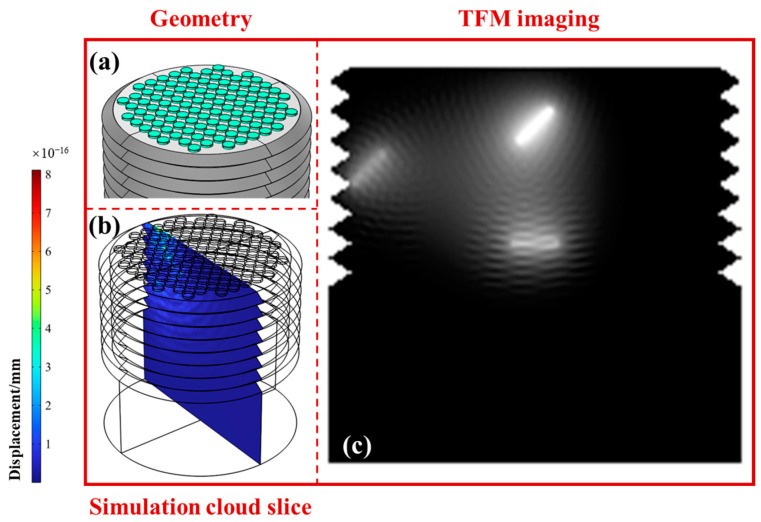
Geometric structure, simulated sound field, and total focusing imaging of the rectangular array ultrasonic phased array. (**a**) Distribution of the array elements; (**b**) corresponding ultrasound field simulation results; (**c**) TFM imaging results.

**Figure 4:**
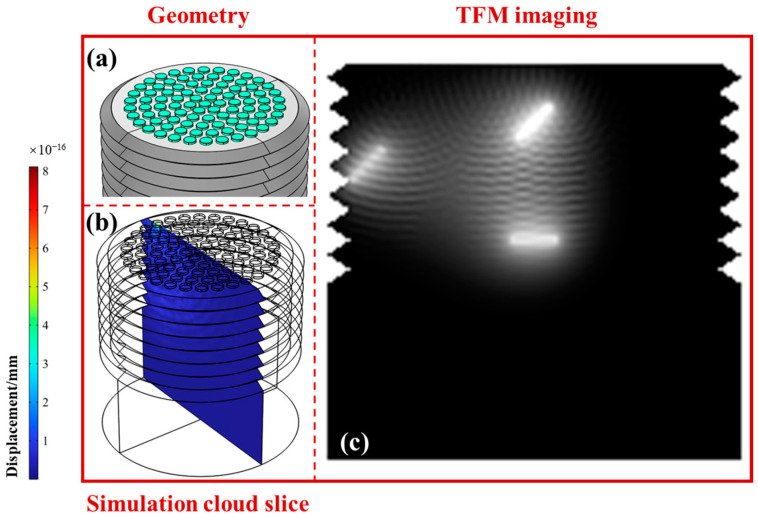
Geometric structure, simulated sound field, and total focusing imaging of the circular array ultrasonic phased array. (**a**) Distribution of the array elements; (**b**) corresponding ultrasound field simulation results; (**c**) TFM imaging results.

**Figure 5:**
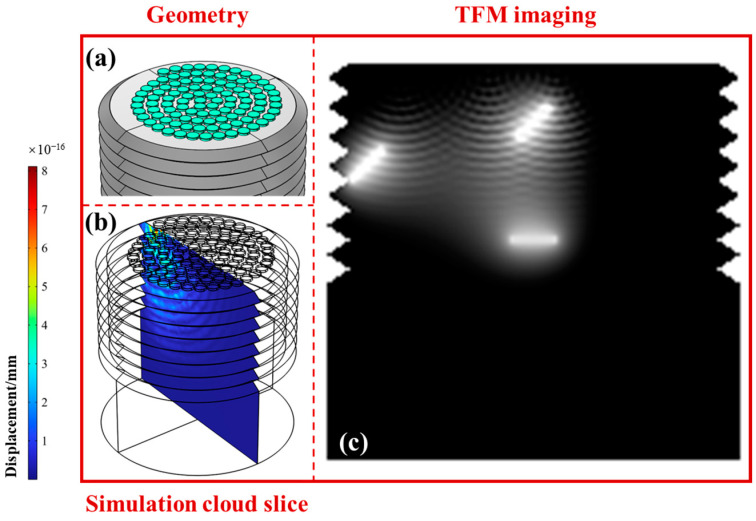
Geometric structure, simulated sound field, and total focusing imaging of the Fermat spiral array ultrasonic phased array. (**a**) Distribution of the array elements; (**b**) corresponding ultrasound field simulation results; (**c**) TFM imaging results.

To verify the superiority of the proposed Fermat spiral ultrasonic phased array, we compared the ultrasonic detection effects of traditional rectangular arrays and circular arrays. The geometric structures of the arrays, the simulated 2D cross-sections, and the TFM imaging results are shown in Figure 3, Figure 4 and Figure 5. The fill rates of the rectangular, circular, and spiral arrays were 49%, 58%, and 87%, respectively, and the normalized amplitudes of the sound fields were 0.24, 0.49, and 1. Here, for the rectangular array, the fill rate was simply the product of element width and height divided by the product of array width and height. For the circular array, the fill rate was calculated as the total area of all circular elements divided by the area of the smallest circle encompassing all the elements. For the Fermat spiral array, the calculation of the fill rate was basically the same as for the circular array. However, special attention must be given to the calculation of the number of elements in the array, as it needs to account for the coefficients of the Fermat spiral line.

Among them, we set three cracks inside the bolt, as shown in Figure 2, located at different positions and with different deflection angles. Generally, crack ① was closest to the probe and had the highest sound field intensity. Crack ②, although farthest from the probe, had a larger reflection cross-section due to its orientation parallel to the r-axis. Crack ③ was located at the root of the thread, where the detection signal was easily interfered with by complex thread stray reflection waves, resulting in poor detection results.

From the simulation results of the rectangular array shown in Figure 3, it can be seen that the ultrasonic sound field of the rectangular array had significant sidelobes and grating lobes. The imaging clarity of crack ① was relatively high, followed by ②, whereas the crack at the root of the thread ③ was almost undetectable. In contrast, the circular array shown in Figure 4 had fewer grating lobes, better imaging effects for cracks ① and ②, and some improvement in the imaging effect for the crack at the root of the thread ③. This is due to the corresponding characteristics of the circular elements and the thread structure. For the Fermat spiral array shown in Figure 5, it can be seen that the sound field amplitude was higher, the grating lobe level was weaker, and the imaging effects for all three cracks were better. The imaging clarity of crack ② was slightly lower than that of ① due to partial signal blocking by crack ①. Therefore, in summary, the spiral array offers significant advantages in the detection and imaging of internal cracks in bolts, with particularly outstanding performance in detecting fatigue cracks at the thread root.

**Figure 6:**
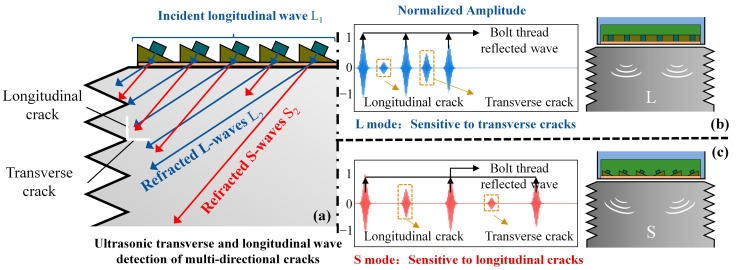
Interaction between S and L ultrasonic modes with longitudinal/lateral cracks, and the simulation signals of thread/crack reflections. (**a**) Schematic diagram of the interaction between different ultrasonic modes and crack defects; (**b**) Simulated defect detection signal for L-mode ultrasound; (**c**) Simulated defect detection signal for S-mode ultrasound.

Additionally, due to the complex propagation directions of bolt cracks, using a single-mode ultrasonic detection method is less effective. Therefore, we adopted a multi-physics dynamic co-simulation method based on COMSOL-MATLAB-SolidWorks (COMSOL 6.1—MATLAB 2022—SolidWorks 2023). This method introduces impedance boundary conditions on the bolt surface and uses coupled solutions of the electromagnetic and solid mechanics modules to construct a three-dimensional general finite element model for the entire ultrasonic spiral phased array transceiver process of wind turbine tower bolts. This approach ultimately allowed us to obtain the coupling characteristics of ultrasonic transverse and longitudinal modes with fatigue cracks in different orientations. The simulation results are shown in Figure 6a–c, where the transmitting elements were upgraded to oblique wedges to excite different ultrasonic modes.

As shown in Figure 6a, there were two cracks at the root of the thread, each 3 mm in length and 0.3 mm in width, extending in longitudinal and lateral directions, respectively. For ultrasonic excitation, the incident wave was longitudinal wave L_1_ and the refracted waves were longitudinal wave L_2_ and transverse wave S_2_, both following Snell’s law in their propagation directions. Figure 6b,c illustrates the ultrasonic response to longitudinal and transverse cracks under L-mode and S-mode excitation, respectively. The characteristics of the reflected waves and their amplitudes have been normalized. From Figure 6b, it can be observed that the amplitude of the reflected wave from the longitudinal crack is lower for the L-mode ultrasonic wave, whereas the signal from the transverse crack is stronger, indicating that the transverse crack reflects more longitudinal wave energy. Similarly, in Figure 6c, the longitudinal crack reflects more transverse wave energy compared to the transverse crack. Therefore, it can be concluded that longitudinal waves are more sensitive to transverse cracks, whereas transverse waves are more sensitive to longitudinal cracks.

**Figure 7:**
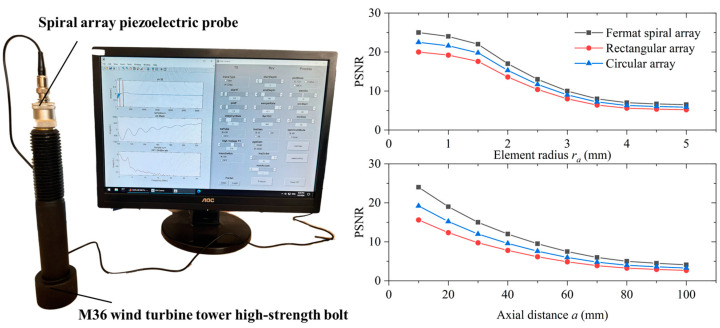
Experimental setup diagram and PSNR results of imaging with varying element radii (*r_a_*) and defect axial distances (*a*).

Figure 7 shows a schematic of the detection of M36 high-strength bolts in a wind turbine tower using a spiral phased array. The ultrasonic excitation and reception device used was the Vantage 128 ultrasonic acquisition system developed by Verasonics, which allows for programmable excitation, reception, and real-time total-focusing imaging of the ultrasonic array through interaction with MATLAB. In addition, it also offers significant enhancements for high-performance applications, particularly in ultrasonic testing for detecting small cracks. It supports the use of high-performance, low-noise connectors, ensuring robust signal integrity that is critical for precise reflection wave analysis. Moreover, it accommodates transducers with up to 128 elements seamlessly, eliminating the need for a multiplexer and enhancing flexibility in configuring various element arrays. The system also includes dedicated signal paths for additional requirements, such as grounding, power supply, and digital control signals, which are essential for comprehensive testing protocols.

In the experiment, using Vantage 128, we analyzed the imaging clarity of crack defects at different element sizes and axial distances, quantified by the peak signal-to-noise ratio (PSNR). The PSNR is an engineering term that represents the ratio of the maximum possible power of a signal to the destructive noise power that affects its precision. Given the wide dynamic range of many signals, the PSNR is often expressed in logarithmic decibels. Figure 7 illustrates the relationship between the element radius (*r_a_*) and the PSNR in total-focusing imaging. The PSNR analysis in Figure 7 specifically pertains to defect ‘③’ shown in Figure 2. The defect was located on the fourth thread root, tilted 45° relative to the r-axis, and measured 3 mm in length and 0.3 mm in width. As the element radius increases, the PSNR initially declines slowly, then more rapidly, and finally slowly again, indicating an overall downward trend. This pattern arises because larger element sizes introduce more interference from stray signals, particularly when the element size significantly exceeds the defect size. We also investigated the performance of different spiral arrays in detecting bolt defects using PSNR. As shown in Figure 7, the Fermat spiral array exhibits the highest PSNR, followed by the circular and rectangular arrays in descending order. This is attributed to the lower sound field intensity and the complexity of sidelobes excited by the circular and rectangular arrays.

Additionally, the figure demonstrates that an increase in the axial distance (*a*) of the defect from the probe correlates with a deterioration in imaging resolution. This reduction in resolution is attributable to the attenuation effect of sound waves during propagation. As sound waves travel further distances, their energy diminishes, leading to a decrease in the clarity and accuracy of the detected signals. Consequently, the larger the axial distance, the poorer the imaging resolution, underscoring the challenges in accurately detecting defects that are farther from the probe.

## 4. Conclusions

The high complexity of thread structures and the high concealment of crack defects in the bolts of wind turbine towers present significant challenges for the online ultrasonic phased array detection of bolt defects. To address the issues of low-energy excitation and poor beam purity in traditional ultrasonic phased arrays, we proposed an ultrasonic phased array structure based on a Fermat spiral circular array. Utilizing the compactness and aperiodicity of ultrasonic elements arranged along the Fermat spirals, we significantly increased the filling rate of the phased array and reduced the grating/sidelobe levels of the beam without increasing the overall size of the phased array. Compared with traditional rectangular and circular arrays, the filling rate of our proposed Fermat spiral ultrasonic array increased by 77.6% and 50%, respectively, and the sound field intensity increased by at least 1-fold. This enables high-sensitivity detection and high-resolution imaging of crack defects in wind turbine tower bolts under high-energy pure sound field excitation using ultrasonic phased arrays.

Furthermore, we found that longitudinal (L-mode) and transverse (S-mode) ultrasonic waves were more sensitive to lateral and longitudinal cracks, respectively. Therefore, future work will further validate the effectiveness of S and L mixed-mode ultrasonic waves in detecting multi-directional cracks in bolts, aiming to achieve rapid and accurate crack detection in various orientations. The computational complexity of the Fermat spiral array design and signal processing, potential challenges in manufacturing spiral arrays with very small element sizes, and the need for further investigation into optimal spiral coefficients for different bolt geometries and crack characteristics all represent significant areas of concern, which will be studied in our future works. Additionally, to ensure the long-term reliable operation of this detection system in the harsh working environment of wind turbines, factors such as system durability, calibration requirements, cost, and resistance to environmental factors need to be considered. Simplifying the calibration process, developing automated calibration software, and implementing online adaptive calibration techniques are important methods for reducing maintenance costs. Future research will explore the use of corrosion-resistant materials, waterproof and dustproof designs, and rigorous environmental testing to enhance system durability.

## Figures and Tables

**Table 1:** Simulation parameters.

	Parameter	Value		Parameter	Value
Bolt	Thread diameter	36 mm	Meshing 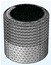	Number of elements	91,547
	Pitch	3 mm		Minimum element quality	0.651
	Bolt length	200 mm		Average element quality	0.994
	Hexagon head height	22 mm		Element volume ratio	1.534 × 10^−7^
	Poisson’s ratio	0.28		Mesh volume	15,680 mm^3^
	Elasticity Modulus	212 GPa	Crack	Length	3 mm
	Hardness	217 HB		Width	0.3 mm
Array	Element radius	1.5 mm	Excitation	Excitation frequency	4 MHz
	Spiral coefficient	3		Excitation voltage	15 V
	Array element number	100		Pulse width	3 μs

## Data Availability

The data that support the findings of this study are available from the corresponding author upon reasonable request.
